# Outcome and prognostic factors of multimodal therapy for pulmonary large-cell neuroendocrine carcinomas

**DOI:** 10.1186/s40001-015-0158-9

**Published:** 2015-08-14

**Authors:** Juliane Rieber, Julian Schmitt, Arne Warth, Thomas Muley, Jutta Kappes, Florian Eichhorn, Hans Hoffmann, Claus Peter Heussel, Thomas Welzel, Jürgen Debus, Michael Thomas, Martin Steins, Stefan Rieken

**Affiliations:** Department of Radiation Oncology, University Hospital Heidelberg, Im Neuenheimer Feld 400, 69120 Heidelberg, Germany; Heidelberg Institute of Radiation Oncology, Heidelberg, Germany; Institute for Pathology, University Hospital Heidelberg, Im Neuenheimer Feld 224, 69120 Heidelberg, Germany; Translational Research Unit, Translational Lung Research Centre Heidelberg (TLRC-H), German Centre for Lung Research (DZL), Im Neuenheimer Feld 430, 69120 Heidelberg, Germany; Department of Thoracic Oncology, Thoraxklinik, Heidelberg University, Amalienstraße 20, 69126 Heidelberg, Germany; Department of Thoracic Surgery, Thoraxklinik, Heidelberg University, Amalienstraße 20, 69126 Heidelberg, Germany; Department of Diagnostic and Interventional Radiology with Nuclear Medicine, Thoraxklinik gGmbH, University Hospital Heidelberg, Heidelberg, Germany; Department of Diagnostic and Interventional Radiology, University Hospital Heidelberg, Heidelberg, Germany

**Keywords:** Lung cancer, Large-cell neuroendocrine carcinoma, Radiotherapy, Brain metastases, Prophylactic cranial irradiation

## Abstract

**Background:**

There is controversy whether patients diagnosed with large-cell neuroendocrine carcinoma (LCNEC) should be treated according to protocols for non-small cell lung cancers (NSCLC) or small cell lung cancers (SCLC), especially with regard to the administration of prophylactic cranial irradiation (PCI). This study was set up to determine the incidence of brain metastases and to investigate the outcome following multimodal treatment in 70 patients with LCNEC.

**Methods:**

Seventy patients with histologically confirmed LCNEC were treated at the University Hospital of Heidelberg between 2001 and 2014. Data were collected retrospectively. Al most all patients received thoracic surgery as initial treatment (94 %). Chemotherapy was administered in 32 patients as part of the initial treatment. Fourteen patients were treated with adjuvant or definitive thoracic radiotherapy according to NSCLC protocols. Cranial radiotherapy due to brain metastases, mostly given as whole brain radiotherapy (WBRT), was received by fourteen patients. Statistical analysis was performed using the long-rank test and the Kaplan–Meier method.

**Results:**

Without PCI, the detected rate for brain metastases was 25 % after a median follow-up time of 23.4 months, which is comparable to NSCLC patients in general. Overall (OS), local (LPFS), brain metastases-free survival (BMFS) and extracranial distant progression-free survival (eDPFS) was 43, 50, 63 and 50 % at 5 years, respectively. Patients with incomplete resection showed a survival benefit from adjuvant radiotherapy. The administration of adjuvant chemotherapy improved the general worse prognosis in higher pathologic stages.

**Conclusion:**

In LCNEC patients, the administration of radiotherapy according to NSCLC guidelines appears reasonable and contributes to acceptable results of multimodal treatment regimes. The low incidence of spontaneous brain metastases questions a possible role of PCI.

## Background

The incidence of large-cell neuroendocrine carcinoma (LCNEC) is low as it accounts for about 3 % of all lung cancer cases [1, 2]. Patients diagnosed with LCNEC suffer from a very dismal prognosis with 5-year overall survival rates between 15 and 57 % [2–5]. During the last years, several reports suggested similarities in histology, clinical behavior and biology of LCNEC and small cell lung cancer (SCLC) [6–9]. Histological differentiation between LCNEC and SCLC can be challenging as both tumor entities often share many common features: neuroendocrine morphology, high mitotic rate, large zones of necrosis and positive immunohistochemical staining for neuroendocrine markers [10, 11]. Furthermore, both LCNEC and SCLC are characterized by common clinical aspects including a predominance of males and smokers and aggressive clinical courses [11–13]. In addition, Jones et al. found comparable genetic alterations in LCNEC and SCLC and were unable to distinguish LCNEC from SCLC by gene expression profiling [14]. However, Ullmann et al. and Hiroshima et al. showed that LCNEC and SCLC harbor distinct morphological, phenotypical and genetical differences [15, 16]. Moreover, analyzing 1,211 patients with LCNEC from the Surveillance, Epidemiology, and End Results (SEER) program of the US National Cancer Institute, Varlotto et al. reported that the clinical, histopathological and biological characteristics of LCNEC were more similar to large-cell carcinoma than to SCLC [17]. Additionally, the World Health Organization still categorizes LCNEC in the group of NSCLC.

Due to the complex clinical, histopathological and biological characteristics of LCNEC, it remains uncertain whether patients diagnosed with LCNEC should be treated according to NSCLC-based or SCLC-based regimes [11, 13, 17]. Current treatment strategies for patients with LCNEC are a mixture between guidelines for NSCLC and SCLC patients: while surgical resection is recommended for all non-metastatic stages analog to NSCLC treatment guidelines, adjuvant chemotherapy (when needed) is administered according to SCLC protocols [13, 18].

In general, treatment strategies also differ strongly between SCLC and NSCLC regarding radiotherapy. Patients diagnosed with SCLC with cN0 and pN1 nodal involvement (limited stage) are treated with thoracic radiotherapy, while patients with NSCLC only benefit from adjuvant radiotherapy in N2 nodal stages [19–21]. Prophylactic cranial irradiation (PCI) is recommended in patients with SCLC, as it prolongs both disease-free and overall survival [22, 23]. On the contrary, PCI is not administered in patients with NSCLC, as Gore et al. only showed a decreased rate of brain metastases after PCI and were not able to detect any significant improvement of overall and disease-free survival after PCI in stage III lung cancer [24]. As treatment strategies vary strongly between SCLC and NSCLC, it is of major interest to find out more about the appropriate treatment regarding radiotherapy for patients with LCNEC.

## Methods

Between 2001 and 2014, seventy patients with histologically confirmed large-cell neuroendocrine carcinoma of the lung were treated at the University Hospital in Heidelberg, Germany. This retrospective analysis was performed with ethical approval by the ethic committee of the University Hospital Heidelberg. Median follow-up time was 23 months (range 0–155 months). One patient was lost to follow-up after 18 months. Detailed patients’ characteristics are shown in Table 1. For tumor grading and staging, the 7th lung cancer TNM classification was used [25, 26]. In our cohort, most patients suffered from locally advanced stages (IIA–IIIB), while stage IV was only detected in seven patients.

**Table 1:** Characteristics of 70 patients diagnosed with large-cell neuroendocrine carcinoma of the lung

Factor	LCNEC (*n* = 70)
Sex	
Male	52 (72 %)
Female	18 (28 %)
Median age (range)	63 years (41–81)
Over 70 years	20 (29 %)
Under 70 years or 70 years	50 (71 %)
TNM stage (7th classification)	
IA/IB	9/10
IIA/IIB	15/9
IIIA/IIIB	18/2
IVA/IVB	7
Thoracic surgery	66 (94 %)
Primary	65 (93 %)
Wedge resection	4 (6 %)
Lobectomy	53 (81 %)
Bilobectomy	3 (5 %)
Pneumonectomy	5 (8 %)
Chemotherapy	36 (51 %)
Definitive	4 (6 %)
Adjuvant	32 (46 %)
Relapse/progress	14 (20 %)
Thoracic radiotherapy	17 (24 %)
Definitive	4 (6 %)
Adjuvant/additive	10 (14 %)
Relapse/progress	3 (4 %)
First line therapy brain metastases	17 (24 %)
Resection	7 (10 %)
Stereotactic irradiation	2 (3 %)
Whole brain irradiation	6 (9 %)
Best supportive care	2 (3 %)
Prophylactic cranial irradiation (PCI)	3 (4 %)

Almost all patients underwent thoracic surgery as part of the initial treatment (93 %) (Table 1). According to current discussions and treatment recommendations, even some patients in oligometastatic tumor stage IV who were in good general health conditions and only suffered from few comorbidities received thoracic surgery [27, 28]. The discussion upon treatment in any case was taken by an interdisciplinary tumorboard conference.

Depending on comorbidity, age and cardiopulmonal function, chemotherapy was mostly administered postoperatively in stage IIA–IIIA according to the German S3-guideline [29]. The types of chemotherapeutic regimes were decided by the treating interdisciplinary team according to the current German S3-guideline at that time (Table 2).

**Table 2:** Different adjuvant chemotherapeutic regimes used in treatment of patients with LCNEC

Chemotherapeutic regime	*N*
SCLC-based regimes	
Cisplatin/etoposide	11
Carboplatin/etoposide	5
NSCLC-based regimes	
Carboplatin/paclitaxel	3
Carboplatin/gemcitabine	1
Carboplatin/vinorelbine	3
Cisplatin/permetrexed	1
Cisplatin/vinorelbine	5
Cisplatin/doxetaxel	2
Gemcitabine	1

Thirty-two patients received adjuvant chemotherapy after thoracic surgery. Of these patients, sixteen patients were treated according to SCLC-based regimes, while the further sixteen patients received chemotherapeutic treatment according to NSCLC-based regimes (Table 2). Stage IV patients without surgical resection were subjected to primary palliative (radio-) chemotherapy. Upon metastatic recurrence, seven patients received palliative chemotherapy.

Postoperative thoracic radiotherapy was administered in N2 stages and incomplete resections (R1 or R2). Doses with 50–60 Gy were used. Patients diagnosed with tumors at higher stages without surgical treatment options were treated with simultaneous radiochemotherapy administering photon doses of 66.0–70.5 Gy.

Two patients already suffered from brain metastases at the date of initial diagnosis, while seventeen patients developed brain metastases during follow-up time. All patients either received contrast-enhanced CT or MRI scan of the brain at initial diagnosis for staging. Before 2010, only CT scans were performed, afterwards 48 % of the patients were subjected to cranial staging with MRI. Treatment for brain metastases comprised whole brain radiotherapy, stereotactic irradiation and surgery. Three patients with locally advanced tumors received (PCI) with a total dose of 30.0 Gy, single dose 2.0 Gy at initial diagnosis of LCNEC. Two of these patients received MRI scans, while one patient was staged using cranial CT due to claustrophobia.

### Statistical analysis

Overall survival (OS) was calculated in months from the date of initial diagnosis until the last date of follow-up or death. Progression-free survival (PFS), local progression-free survival (LPFS), brain metastases-free survival (BMFS) and extracranial distant progression-free survival (DPFS), as well as brain metastases-free survival, were calculated from the date of primary diagnosis until the first imaging diagnosis of recurrent disease. Death due to any cause or lost to follow-up (one patient) was taken as an event. Data analysis was censored as not all patients suffered from an event during follow-up time. The Kaplan–Meier method was used to display results. Survival curves were compared between groups in a univariate analysis using the long-rank test. All statistical analyses were performed with a software tool (SPSS 20.0).

## Results

**Fig. 1:**
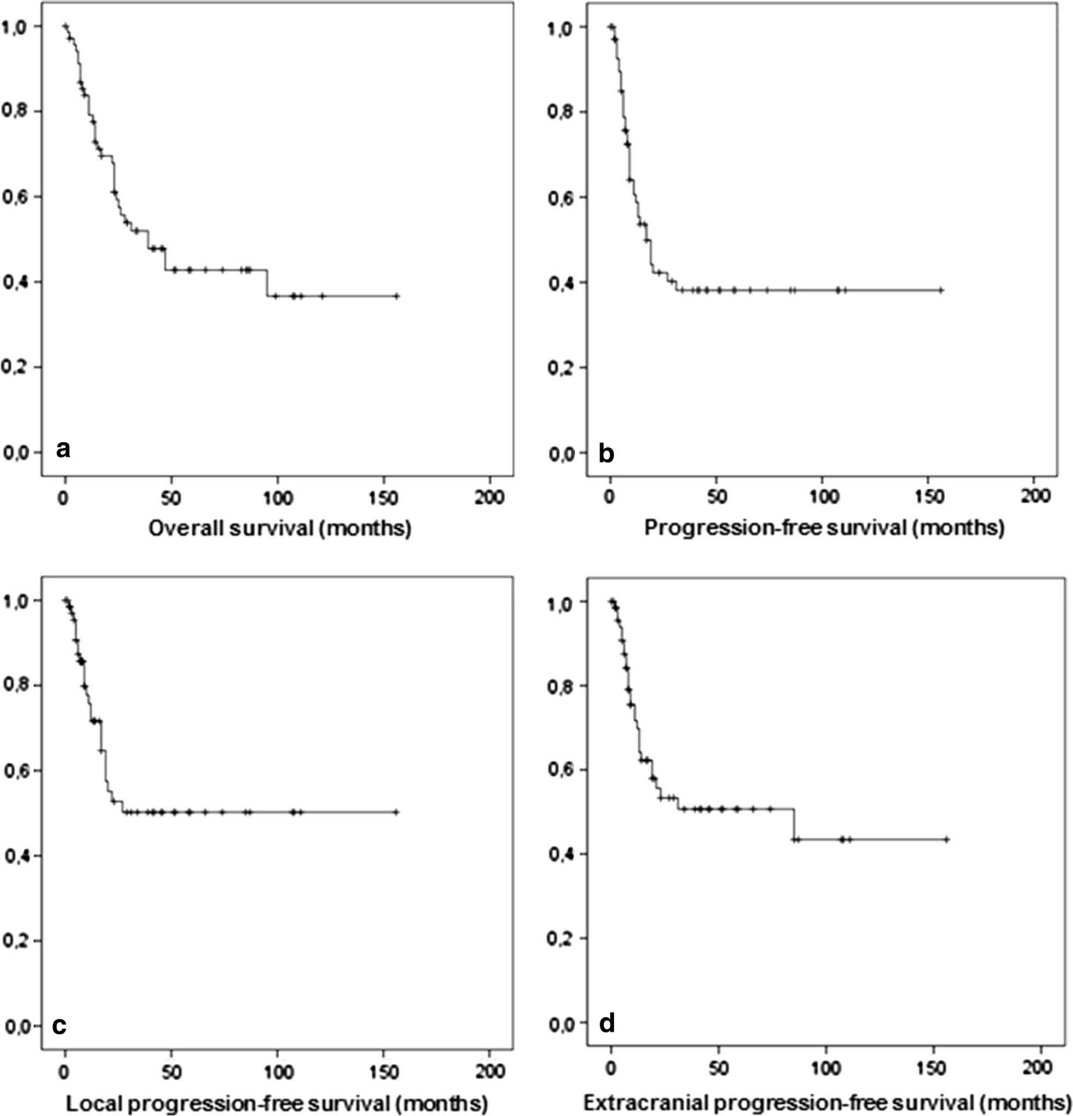
Overall (**a**) and progression-free (**b**), local (**c**) and extracranial distant progression-free survival (**d**) in 70 patients with LCNEC.

The follow-up period for the patients in this study ranged from 0 months to 155 months. The median follow-up time was 23 months. The survival curve for the seventy patients with LCNEC is shown in Fig. 1a. The 2-year and the 5-year overall survival rates (OS) were 74 and 43 %, respectively. Survival was dependent on tumor stage: patients with stage I–II LCNEC showed 2- and 5-year overall survival rates of 67 and 48 %, while patients diagnosed with stage III–IV LCNEC had a 2- and 5-year overall survival of 39 and 29 %. 2- and 5-year progression-free survival rates (PFS) were 74 and 38 %, respectively (Fig. 1b). Local progression-free survival was 75 % after 2 years and 50 % after 5 years (Fig. 1c). 2- and 5-year extracranial distant progression-free survival was 53 and 50 %, respectively (Fig. 1d).

### Brain metastases

**Fig. 2:**
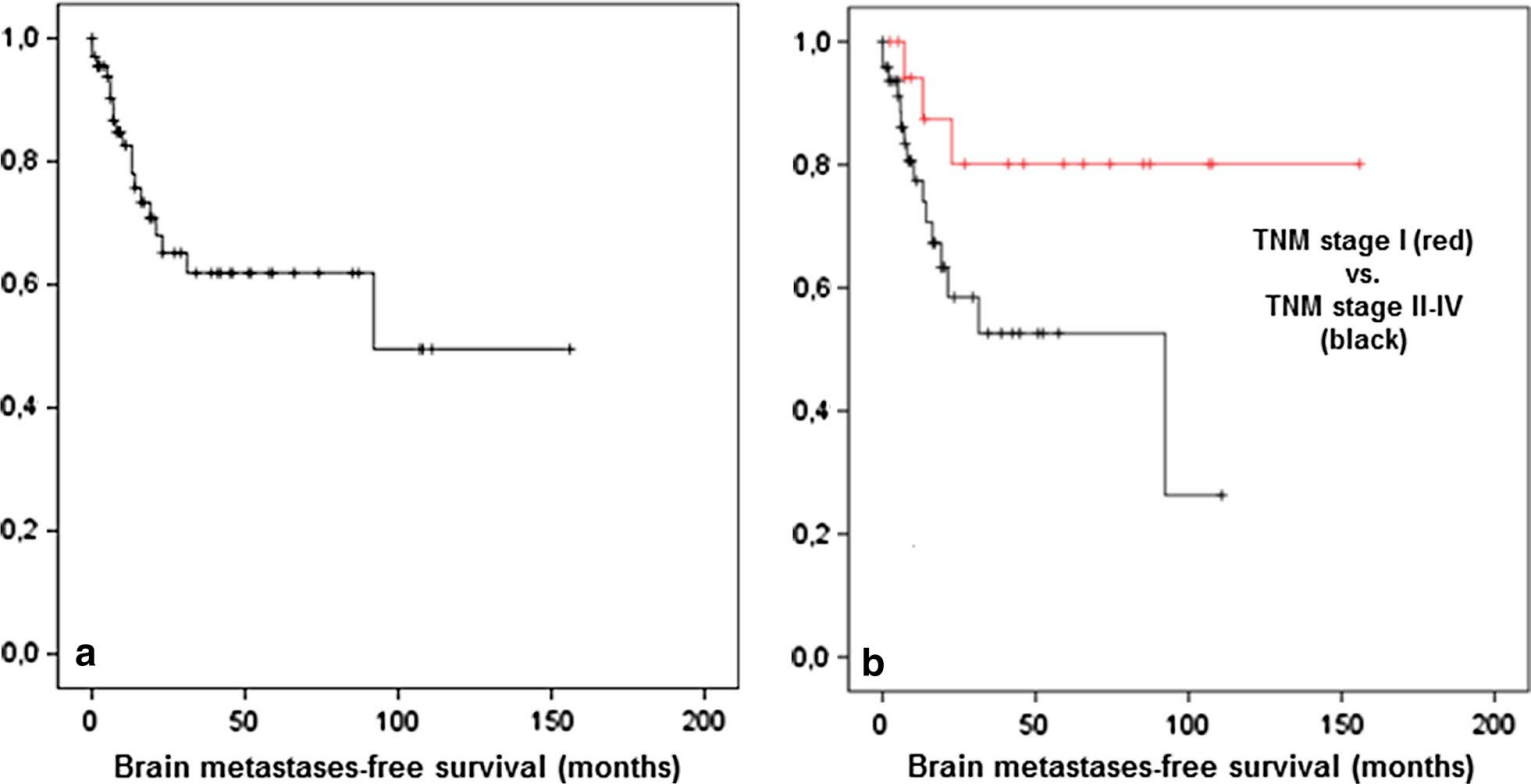
Brain metastases-free survival (**a**) is significantly dependent on tumor stages (**b**) (p = 0.045).

Seventeen patients (25 %) were diagnosed with brain metastases during follow-up. While 9 patients only suffered from 1 to 2 brain metastases, 8 patients presented with disseminated metastases (more than three). Patients presenting with 1–2 metastases either received surgical resection or radiosurgery. Hence, brain metastases-free survival was 85 % after 2 years and 63 % after 5 years (Fig. 2a). Brain metastases-free survival was not influenced by age and sex. Interestingly, the development of brain metastases was significantly associated with pathologic stage when comparing stage I vs. stage II–IV tumors (*p* = 0.045; Fig. 2b).

Patients with brain metastases suffered from significantly reduced overall survival with 2- and 5-year rates of 37 and 16 %, while patients without brain metastases showed 2- and 5-year overall survival rates of 69 and 54 %, respectively (*p* = 0.002). Along with the introduction of SCLC-derived chemotherapy regimes, three patients with advanced tumor stages received prophylactic cranial irradiation (PCI). Patients with PCI did not develop brain metastases during follow-up time (8.2 months). During follow-up time, 37 (53 %) LCNEC patients developed progression with local relapse in 25 patients (36 %) and distant progression in 28 patients (40 %). First relapse was distant in 6 (16 %) and combined local and distant at the same time in 31 patients (83.4 %).

Apart from the patients with cerebral metastases, 17 patients (24 %) developed distant metastases: liver (7), bone (7), adrenal (4) and lung (2).

### Treatment concepts

**Fig. 3:**
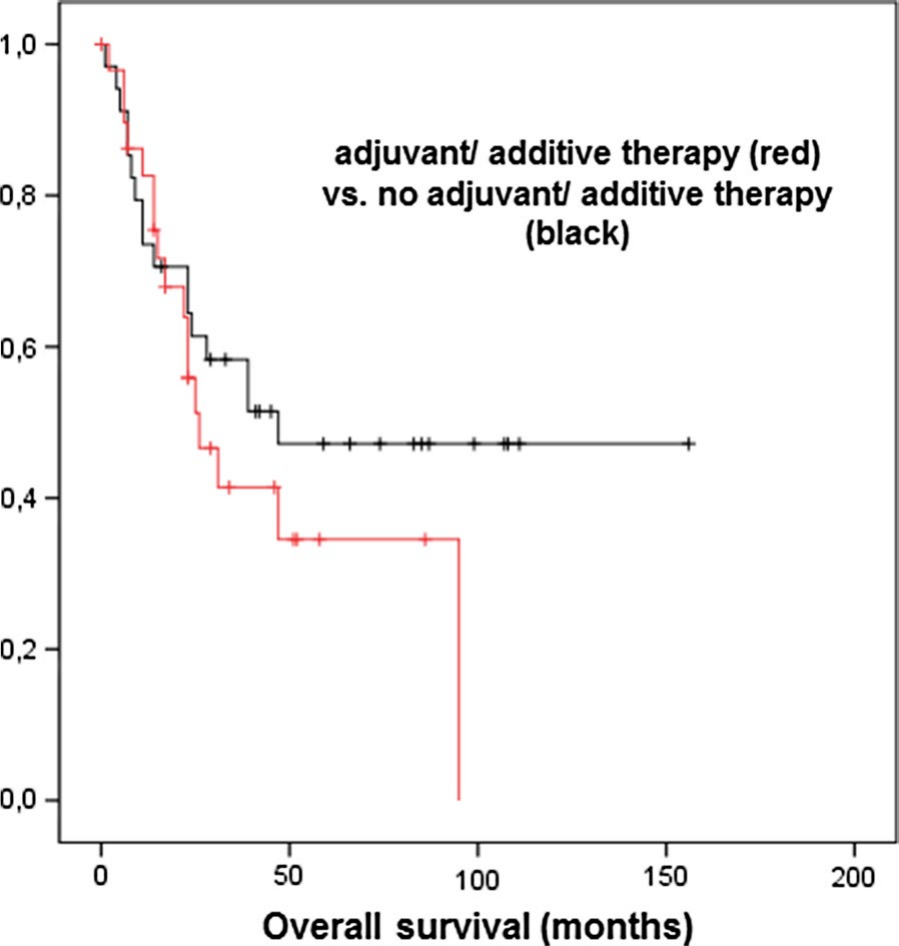
Adjuvant chemotherapy in higher pathologic stages improves overall survival.

Patients with incompletely resected LCNEC tumors received postoperative radiotherapy and showed non-inferior survival rates with 2- and 5-year overall survival of 50 and 30 %, respectively (*p* = 0.89). In addition, we analyzed the different treatment concepts regarding overall and disease-free survival. Chemotherapy was administered in heterogeneous regimes (Table 2). According to the German S3-guideline, patients in higher stages were treated with adjuvant chemo-, radio- or radiochemotherapy after primary resection [29]. Regarding overall survival, we compared patients who only received resection as primary treatment (*n* = 34; 53 %) to the patients who were treated with resection and adjuvant chemo-, radio- or radiochemotherapy, mainly because of higher tumor stages (≥IIIA) (*n* = 30; 47 %). Both groups did not differ in overall survival (*p* = 0.298) (Fig. 3). Additionally, local progression-free survival was also not significantly different between these two groups (*p* = 0.412). Furthermore, comparing chemotherapy regimes derived from SCLC vs. NSCLC protocols, we did not detect a difference in treatment results and patient outcome.

## Discussion

In the present study, we evaluated incidence of brain metastases, treatment outcome and prognostic factors in 70 patients with LCNEC who received multimodal treatment. In our cohort, 17 patients (25 %) were diagnosed with brain metastases during follow-up of 2 years. In NSCLC, 20–30 % of patients are diagnosed with brain metastases, while in SCLC already 50 % of patients suffer from brain metastases 2 years after initial diagnosis [22, 30, 31]. Due to the complex clinicopathological and biological features of patients with LCNEC, there is no consensus on whether LCNEC should be treated according to SCLC or NSCLC protocols [13]. Among others, Sun et al. claimed that LCNEC should receive treatment similar to SCLC, while Varlotto et al. insisted on treating LCNEC with NSCLC treatment regimes [11, 17, 32]. As Aupérin et al. showed that prophylactic cranial irradiation (PCI) improves both overall and disease-free survival among patients with limited disease SCLC in complete remission, PCI became standard treatment in patients with limited disease SCLC [22, 29]. Some years later, Slotman et al. established PCI for patients with extensive disease SCLC [23]. Regarding patients diagnosed with NSCLC, prophylactic cranial irradiation is usually not recommended in higher pathologic stages. Gore et al. only detected a decreased rate of brain metastases after PCI and were not able to show any significant improvement of overall or disease-free survival after PCI in stage III lung cancer [24, 29]. Nevertheless, Iyoda et al. proposed that PCI might be promising for patients with LCNEC [33]. Indeed, the three patients who received PCI in our cohort did not develop brain metastases during follow-up time (8.23 months). However, in our study 25 % of patients developed brain metastases during follow-up which is comparable to patients with NSCLC and not to patients with SCLC. Similar to our results, Iyoda et al. showed that about 19 % of the analyzed LCNEC patients developed brain metastases [32, 34]. Interestingly, the development of brain metastases was significantly associated with pathologic TNM stage in our cohort. We detected a continuous, non-saturable but shallow decline in brain metastases-free survival. In general, patients with SCLC in lower stages tend to show the greatest benefit from PCI [22]. As on the one hand, only few LCNEC patients develop brain metastases and on the other hand, there was a strong correlation between pathologic stage and the development of brain metastases in our study, prophylactic cranial irradiation (PCI) in patients with LCNEC should be reconsidered thoroughly, especially in lower tumors stages.

LCNEC used to be only treated by resection in all tumor stages and therefore showed poor survival rates [1, 35, 36]. Adjuvant therapy, mainly chemotherapy, led to a subsequent improvement in survival in patients with higher tumor stages [5, 37, 38]. In NSCLC, adjuvant thoracic radiotherapy is only recommended in pN2 and higher nodal stages and after incomplete resection. On the contrary, patients with SCLC benefit from thoracic radiotherapy when diagnosed with cN0 and pN1 nodal involvement (limited stage) [19–21, 29]. LCNEC tumors appear to be radiation responsive as patients with incompletely resected LCNEC and adjuvant radiotherapy showed non-inferior survival. However, excellent local control was detected in patients with limited thoracic tumor extension. Hence, radiotherapy is not needed in lower tumor stages and should only be applied according to NSCLC treatment protocols.

Sarkaria et al. reported that LCNEC had a high response rate to platinum-based chemotherapy. Furthermore, resected advanced stage patients receiving combination neoadjuvant and (or) adjuvant chemotherapy tended to have a survival benefit [39]. Correspondently, additional treatment with adjuvant or additive chemo-, radio-, or radiochemotherapy in higher stage large-cell neuroendocrine carcinoma led to overall survival rates comparable to patients who underwent surgery only for low-stage NSCLC. This fact underlines the importance of adjuvant treatment in LCNEC regarding overall survival. Sun et al. revealed that the response rate to platinum-based chemotherapy was 60 % in LCNEC patients, whereas the response rate of non-platinum-based chemotherapy was only 11 % [11]. Interestingly, all but one patient in our cohort received adjuvant platinum-based chemotherapy which might have led to the improved survival rates. Yamazaki et al. also reported a similar response rate to platinum-based chemotherapy and suggested that the response rate of LCNEC patients was comparable to that of SCLC patients [40]. Due to the good response rate to platinum-based chemotherapy and the partly comparable histopathological features of LCNEC and SCLC, mainly chemotherapeutic regimes used in the treatment of SCLC were evaluated within the last years [10].

In our cohort, treatment results and patient outcome was not significantly different comparing chemotherapy regimes derived from SCLC versus NSCLC patients. This may be related to platinum administration in both SCLC and NSCLC regimes in our cohort (Table 2). Rossi et al. analyzed 83 patients with LCNEC and showed that SCLC-based chemotherapy (platinum-etoposide) was an important variable significantly correlating with survival, both in adjuvant and metastatic settings [41]. Iyoda et al. started a prospective trial of adjuvant chemotherapy with platinum and etoposide and reported that SCLC-based chemotherapy appeared promising for the improvement of overall survival in LCNEC patients [42]. Similarly, Treut et al. conducted a multicenter phase II study and found that the outcomes of advanced LCNEC treated with cisplatin and etoposide were comparable to that of advanced SCLC [43]. Recently, a phase III trial comparing irinotecan and cisplatin with etoposide and cisplatin in adjuvant chemotherapy for completely resected LCNEC was initiated [44].

## Conclusion

Despite poor survival rates, only 25 % of LCNEC patients developed brain metastases and therefore, PCI should not be considered a standard component of LCNEC treatment regimes. Adjuvant or additive therapy according to the current German S3-guideline in higher stage large-cell neuroendocrine carcinoma led to overall survival rates comparable to patients who underwent surgery only for low-stage NSCLC, putting emphasis on the need for most effective first-line treatments. Despite its rare occurrence, prospective multi-center trials are needed to truly evaluate the optimal multi-modal therapy for patients with LCNEC.
